# Response to different furosemide doses predicts AKI progression in ICU patients with elevated plasma NGAL levels

**DOI:** 10.1186/s13613-018-0355-0

**Published:** 2018-01-17

**Authors:** Ryo Matsuura, Yohei Komaru, Yoshihisa Miyamoto, Teruhiko Yoshida, Kohei Yoshimoto, Rei Isshiki, Kengo Mayumi, Tetsushi Yamashita, Yoshifumi Hamasaki, Masaomi Nangaku, Eisei Noiri, Naoto Morimura, Kent Doi

**Affiliations:** 1 Department of Nephrology and Endocrinology, The University of Tokyo Hospital, 7-3-1 Hongo, Bunkyo-ku, Tokyo, 113-8655 Japan; 2 Department of Emergency and Critical Care Medicine, The University of Tokyo Hospital, 7-3-1 Hongo, Bunkyo-ku, Tokyo, 113-8655 Japan; 3 Department of Dialysis and Apheresis, The University of Tokyo Hospital, 7-3-1 Hongo, Bunkyo-ku, Tokyo, 113-8655 Japan

**Keywords:** Acute kidney injury, Biomarkers, Intensive care unit, Progression, Diuretics

## Abstract

**Background:**

Furosemide responsiveness (FR) is determined by urine output after furosemide administration and has recently been evaluated as a furosemide stress test (FST) for predicting severe acute kidney injury (AKI) progression. Although a standardized furosemide dose is required for FST, variable dosing is typically employed based on illness severity, including renal dysfunction in the clinical setting. This study aimed to evaluate whether FR with different furosemide doses can predict AKI progression. We further evaluated the combination of an AKI biomarker, plasma neutrophil gelatinase-associated lipocalin (NGAL), and FR for predicting AKI progression.

**Results:**

We retrospectively analyzed 95 patients who were treated with bolus furosemide in our medical–surgical intensive care unit. Patients who had already developed AKI stage 3 were excluded. A total of 18 patients developed AKI stage 3 within 1 week. Receiver operating curve analysis revealed that the area under the curve (AUC) values of FR and plasma NGAL were 0.87 (0.73–0.94) and 0.80 (0.67–0.88) for AKI progression, respectively. When plasma NGAL level was < 142 ng/mL, only one patient developed stage 3 AKI, indicating that plasma NGAL measurements were sufficient to predict AKI progression. We further evaluated the performance of FR in 51 patients with plasma NGAL levels > 142 ng/mL. FR was associated with AUC of 0.84 (0.67–0.94) for AKI progression in this population with high NGAL levels.

**Conclusions:**

Although different variable doses of furosemide were administered, FR revealed favorable efficacy for predicting AKI progression even in patients with high plasma NGAL levels. This suggests that a combination of FR and biomarkers can stratify the risk of AKI progression in a clinical setting.

**Electronic supplementary material:**

The online version of this article (10.1186/s13613-018-0355-0) contains supplementary material, which is available to authorized users.

## Background

Acute kidney injury (AKI) is highly prevalent in an intensive care unit (ICU) and is associated with significant morbidity and mortality [1–3]. Severe AKI has an unacceptably high mortality, especially when renal replacement therapy (RRT) is required [4–6]. Prediction of AKI progression from a mild to severe form is clinically important for several reasons. First, the early initiation of RRT can be supported for highly possible AKI progression, although there is currently no consensus regarding the timing of initiating RRT [7, 8]. Second, AKI diagnosis is based on the changes of serum creatinine concentration, but it is well known that changes in serum creatinine levels are delayed. Although the Kidney Disease: Improving Global Outcomes (KDIGO) Clinical Practice Guideline for Acute Kidney Injury suggests considering an invasive diagnostic workup (stage 1) along with ICU admission (stage 2) for AKI management based on AKI severity determined by serum creatinine [9], establishing a triage decision for management and prevention of AKI progression is difficult with a late marker of serum creatinine. Finally, identifying possible AKI progressors may contribute to the development of novel drugs for AKI by reducing inappropriate enrollment of patients with mild AKI who recover spontaneously.

To date, multiple biomarkers, such as plasma neutrophil gelatinase-associated lipocalin (NGAL), L-type fatty acid binding protein (L-FABP), interleukin (IL)-18, and tissue inhibitor of metalloproteinases (TIMP-2)/insulin-like growth factor-binding protein 7 (IGFBP7), have been developed [10–14]. Moreover, urinary NGAL and L-FABP can reportedly discriminate between prerenal and renal AKI [15–17] and TIMP-2/IGFBP7 can predict AKI progression [18, 19].

Furosemide is excreted from the blood into the urine through the proximal tubules by the human organic anion transporter and inhibits luminal sodium transporters in the loop of Henle from the urinal lumen [20]. If furosemide administration increases the urine output, it could be assumed that the tubules are functional. Koyner et al. [21] recently demonstrated that the 2-h urine output after a standardized high-dose intravenous furosemide injection (furosemide stress test; FST) was sensitive in predicting AKI progression to stage 3 in patients with early AKI.

To better stratify the risk of AKI progression, a combination of renal functional and damage biomarkers is recommended [22]. However, there are no reports in the literature that have examined the combination of functional and damage biomarkers for predicting AKI progression. In this study, we retrospectively evaluated the combination of AKI biomarkers and urine output in response to the administration of bolus furosemide for stratifying the risk of AKI progression in critically ill patients.

## Methods

### Definition

Furosemide responsiveness (FR) is newly defined as total urine output in 2 h (mL) divided by the dose of bolus furosemide (mg) administered. A previous study reported that the urine output within the first 2 h after a standardized dose of furosemide administration provided the highest prediction of the development of severe AKI [23]. We reviewed furosemide dose and hourly urine output using ICU medical charts and determined FR of each patient. The timing and dose of furosemide administration were determined by the physician involved. In our regular clinical practice, furosemide dose was decided based on body weight, volume status, cardiac function, serum creatinine concentration at the time of furosemide administration, and presence of complications of chronic kidney disease (CKD). All patients finally enrolled in this study had an indwelling catheter, and hourly urine output could be accurately measured.

### Study design

**Fig. 1:**
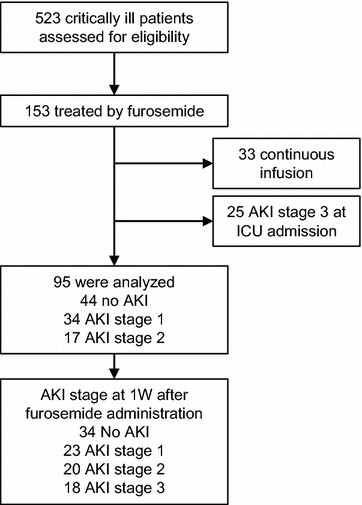
Study flow diagram

This study is a subanalysis of our prospective observational studies [24–26]. The cohort in this study was selected from these previous prospective observational studies conducted in the medical–surgical mixed ICU at the University of Tokyo Hospital. In previous studies, we measured the AKI biomarkers of plasma NGAL, urinary L-FABP, and urinary N-acetyl-β-d-glucosaminidase (NAG) and evaluated the association with AKI biomarkers and AKI progression within 1 week. Among 523 adult critically ill patients enrolled, 153 were retrospectively identified to have received furosemide on the same day that the above-mentioned AKI biomarkers were measured. Finally, 95 patients were eligible for analysis after excluding 33 patients who were administered continuous intravenous furosemide infusion instead of a bolus and 25 patients who had already progressed to AKI stage 3 at the time of ICU admission (Fig. 1). Volume depletion was evaluated by the clinical context, physical signs, and findings on cardiac ultrasound examination in all 95 patients. The study protocol was approved by the institutional review board of the University of Tokyo and adhered to the Declaration of Helsinki. Patient informed consent was obtained at the time of ICU admission. The following clinical variables during the ICU and hospital stay were evaluated: age, sex, weight, causes of ICU admission, acute physiology and chronic health evaluation II score [27], and the length of ICU and hospital stay.

### Assessment of kidney function

Baseline serum creatinine level was defined as the last outpatient value within 6 months prior to ICU admission. If the creatinine level prior to admission was not known, the baseline value was defined as the lowest among creatinine values in hospital but prior to ICU admission, the last level before hospital discharge, and the estimated value using the Modification of Diet in Renal Disease equation at the lower end of the normal range [28]. The definition and classification of AKI were made according to the KDIGO Clinical Practice Guideline for Acute Kidney Injury [29].

### Measurement of AKI biomarkers

Urine and plasma samples were collected at the time of ICU admission and were frozen at − 80 °C within 1 h of collection. As urine output was measured hourly by an indwelling catheter, we could obtain a fresh urine sample that was collected within 1 h previously. The plasma NGAL level was determined using the Triage NGAL Device (AlereMedical, San Diego, CA, USA). Urinary L-FABP level was measured using commercially available enzyme-linked immunosorbent assay kits (Human L-FABP Assay Kit; CMIC Co. Ltd., Tokyo, Japan). Urinary NAG level was measured at the University of Tokyo Hospital Clinical Laboratory using the 4-HP-NAG substrate method (L-Type NAG; Wako Pure Chemical Industries Ltd., Osaka, Japan). Urinary L-FABP and NAG level measurements were evaluated by adjusting with urine creatinine concentration [11, 30].

### Statistical analyses

Data are presented as median (interquartile range). Continuous variables were compared using Wilcoxon rank-sum tests if they were non-normally distributed. Categorical variables were compared using the Pearson Chi-square or Fisher exact test. The urinary and plasma biomarker performance was ascertained using a receiver operating characteristic (ROC) curve analysis. The optimal cutoff values were acquired using the Youden index (sensitivity + specificity − 1), which is a common summary measure of the ROC curve representing the maximum potential effectiveness of a marker [31]. Comparisons of the ROC curves were performed as previously reported [30, 32]. All analyses were performed using a statistical analysis software (JMP ver. 11.2; SAS Institute Inc., Cary, NC). A conventional criterion of an *α* level of 0.05 was used to assess statistical significance.

## Results

### Patient characteristics and AKI progression to AKI stage 3

Characteristics of all 95 patients studied are presented in Table 1. Among these, 51 patients (54%) were diagnosed with AKI at the time of furosemide administration; 34 were diagnosed with AKI stage 1 (36%) and 17 with AKI stage 2 (18%). Within 1 week following furosemide administration, 18 patients progressed to AKI stage 3 (Fig. 1). Among this, 10 patients had progressed from AKI stage 1 and four patients from stage 2. Four patients did not have AKI at the time of furosemide administration.

**Table 1:** Characteristics of the 95 enrolled patients

Age	67 (57–77)
Male/female	58/37
Body weight (kg)	59.7 (48.7–66.6)
APACHE II score	17 (14–22)
Chronic heart failure	13
Furosemide use before ICU	25
Doses of furosemide before ICU (mg)	30 (20–55)
Indication for ICU admission	
Cardiovascular	11
Cerebrovascular	12
Pulmonary	17
Sepsis	17
Others	38
Baseline serum creatinine (mg/dl)	0.8 (0.51–0.98)
Serum creatinine at hospitalization (mg/dl)	0.89 (0.64–1.30)
Serum creatinine at ICU admission (mg/dl)	0.97 (0.60–1.37)
Serum creatinine at furosemide administration (mg/dl)	1.09 (0.68–1.40)
Serum albumin at furosemide administration (g/dL)	2.9 (2.5–3.2)
AKI stage at ICU admission	
No AKI	55
Stage 1	29
Stage 2	11
AKI stage at furosemide administration	
No AKI	44
Stage 1	34
Stage 2	17
AKI stage at 1 week	
No AKI	34
Stage 1	23
Stage 2	20
Stage 3	18
Length of hospitalization (days)	50 (25–93)
Length of ICU stay (days)	6 (3–11)
Plasma NGAL at furosemide administration (ng/mL)	147 (78–309)
Urinary L-FABP at furosemide administration (μg/gCr)	39.9 (15.1–208)
Urinary NAG at furosemide administration (U/gCr)	3.5 (2.0–6.4)
Dose of furosemide (mg)	10 (10–20)

*AKI* acute kidney injury, *APACHE* acute physiology and chronic health evaluation, *ICU* intensive care units, *L-FABP* L-type fatty acid binding protein, *NAG N*-acetyl-β-d-glucosaminidase, *NGAL* neutrophil gelatinase-associated lipocalin

### Biomarkers, FR, and AKI stages at 1 week

**Fig. 2:**
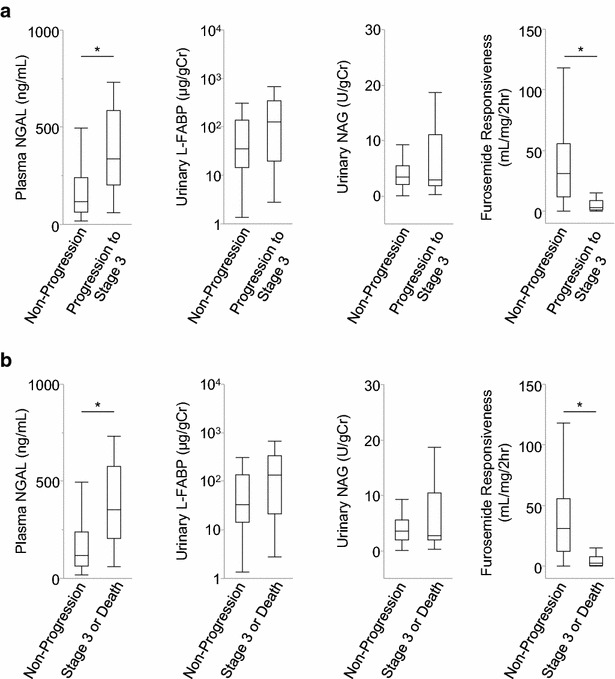
Biomarkers and furosemide responsiveness (FR) in AKI progression. The boxplots show the differences in the AKI biomarkers and FR between patients **a** without and with the progression to AKI stage 3 and **b** without and with the progression to AKI stage 3 or death within 1 week. *p < 0.01

**Fig. 3:**
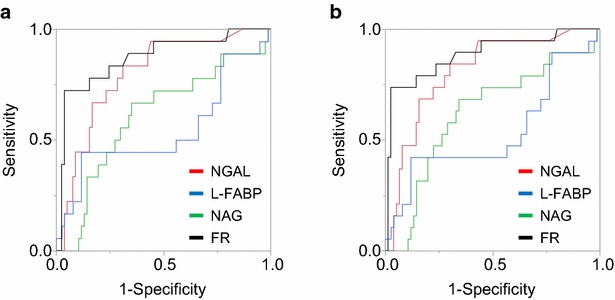
Prediction of AKI progression by biomarkers and furosemide responsiveness (FR). Receiver operating characteristic curves (ROC) in **a** progression to AKI stage 3 and **b** progression to AKI stage 3 or death at 1 week. NGAL, neutrophil gelatinase-associated lipocalin; L-FABP, L-type fatty acid binding protein; NAG, *N*-acetyl-β-d-glucosaminidase; FR, furosemide responsiveness

First, the association between progression to AKI stage 3 after 1 week with FR and AKI biomarkers was evaluated. FR and plasma NGAL level showed significant differences between the AKI progression group (from any stage to stage 3) and the non-progression group (Fig. 2). When weight-adjusted FR is defined as the total urine output in 2 h divided by furosemide dose per kilogram body weight (mg/kg), weight-adjusted FR showed a significant difference between the groups (Additional file 1: Figure S1). Measurement of urinary L-FABP and NAG levels could not significantly differentiate AKI progression to stage 3. The ROC analysis demonstrated that FR, weight-adjusted FR, and plasma NGAL could significantly predict AKI progression to stage 3; in contrast, urinary L-FABP and NAG levels could not predict AKI progression (Table 2). Similar results were obtained when a composite outcome of AKI stage 3 or death within 1 week after furosemide administration was used (Table 2; Figs. 2, 3; Additional file 1: Figure S2).

**Table 2:** ROC analysis for the progression to AKI stage 3 or death within 1 week

	AUC (95% CI)	Cutoff
Progression to AKI stage 3		
FR	0.87 (0.73–0.94)	3.9 mL/mg/2 h
Plasma NGAL	0.80 (0.67–0.88)	199 ng/mL
Urinary L-FABP	0.61 (0.45–0.75)	83.0 mg/μCr
Urinary NAG	0.54 (0.37–0.71)	9.3 U/gCr
Progression to AKI stage 3 or death within 1 week		
FR	0.88 (0.75–0.95)	3.9 mL/mg/2 h
Plasma NGAL	0.81 (0.68–0.89)	199 ng/mL
Urinary L-FABP	0.62 (0.47–0.76)	83.0 mg/μCr
Urinary NAG	0.53 (0.37–0.69)	9.3 U/gCr

*AKI* acute kidney injury, *FR* furosemide responsiveness, *L-FABP* L-type fatty acid binding protein, *NAG N*-acetyl-β-d-glucosaminidase, *NGAL* neutrophil gelatinase-associated lipocalin

### FR for the prediction of AKI progression in the population with high NGAL levels

**Fig. 4:**
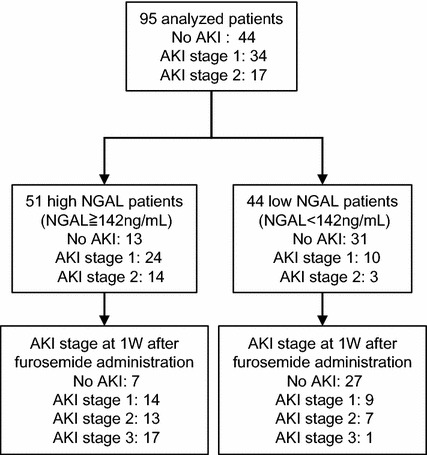
Distribution of ICU patients determined by the plasma NGAL level. NGAL, neutrophil gelatinase-associated lipocalin

Among the 95 enrolled patients, 51 were diagnosed with AKI of different stages at the time of furosemide administration. For predicting AKI progression to stage 3, the plasma NGAL level measured at the time of furosemide administration showed a good AUC in the ROC analysis, 0.79 (0.68–0.86) with a cutoff value of 142 ng/mL, as determined by the Youden index (sensitivity, 72.1%; specificity, 79.4%). When the plasma NGAL level was < 142 ng/mL, only one patient progressed to AKI stage 3, indicating that plasma NGAL level alone was sufficient to predict AKI progression to stage 3. Therefore, we further evaluated the efficacy of FR in predicting AKI progression in patients with plasma NGAL levels > 142 ng/mL. Among the 51 patients with plasma NGAL levels > 142 ng/mL at the time of furosemide administration, 17 progressed to AKI stage 3 (eight patients required RRT) and four died (Fig. 4). FR was associated with AUCs of 0.84 (0.67–0.94) and 0.88 (0.70–0.96) to predict the development of AKI stage 3 and the composite outcome of AKI stage 3 progression or death. Cutoff values of FR for both AKI progression to stage 3 and the composite outcome as determined by Youden index were 3.9 mL/mg/2 h.

The characteristics of the population with higher NGAL levels divided by FR positive (*n* = 36) or FR negative (*n* = 15) with the cutoff value of 3.9 mL/mg/2 h described above are shown in Table 3. The serum creatinine levels at the time of hospitalization and furosemide administration were higher in the FR-positive patients compared with the FR-negative patients. The proportion of patients at each stage of AKI at the time of ICU admission and furosemide administration, as well as the plasma NGAL level measured at the time of furosemide administration, were not significantly different. A higher dose of furosemide was administered to patients who were FR negative than to those who were FR positive. Among the 15 patients who were FR negative, 13 (86.7%) progressed to AKI stage 3, while six (40%) required RRT. On the other hand, among 36 patients who were FR positive, only four (11%) progressed to AKI stage 3 and two (5.6%) required RRT (Table 4).

**Table 3:** Characteristics of patients with high plasma NGAL levels

	All (*N* = 51)	FR positive (*N* = 36)	FR negative (*N* = 15)	P value
Age	65 (55–76)	63.5 (49–73)	73 (61–78)	0.08
Male/female	34/17	24/12	10/5	1.00
APACHE II score	20 (14–22)	19.5 (15–22)	20 (14–28)	0.56
Chronic heart failure	10	5	5	0.12
Furosemide use before ICU	18	10	8	0.08
Dose of furosemide before ICU (mg)	20 (15–40)	40 (15–60)	20 (12.5–40)	0.52
Serum creatinine (mg/dl)				
Baseline	0.83 (0.51–1.36)	0.81 (0.49–1.13)	1.24 (0.8–2.59)	0.07
At hospitalization	1.1 (0.78–1.92)	0.97 (0.69–1.7)	1.81 (0.96–3.64)	0.04
At ICU admission	1.26 (0.92–1.94)	1.13 (0.78–1.48)	1.56 (1.01–2.14)	0.08
At furosemide administration	1.33 (0.99–1.94)	1.17 (0.98–1.67)	1.77 (1.26–2.77)	0.03
Serum albumin (g/dL)	2.8 (2.3–3.1)	2.8 (2.3–3.1)	2.7 (2.3–3.2)	0.79
AKI stage at ICU admission				0.92
No AKI	20	15	5	
Stage 1	21	14	7	
Stage 2	10	7	3	
AKI stage at furosemide administration				0.80
No AKI	13	10	3	
Stage 1	24	16	8	
Stage 2	14	10	4	
Length of hospitalization (days)	42 (25–85)	45 (26–90)	33 (17–77)	0.23
Length of ICU stays (days)	6 (3–13)	5 (3–9)	12 (4–16)	0.04
Plasma NGAL at furosemide administration (ng/ml)	303 (199–495)	274 (190–399)	354 (260–576)	0.26
Dose of furosemide (mg)	20 (10–20)	10 (10–20)	40 (20–45)	< 0.01

*AKI* acute kidney injury, *APACHE* acute physiology and chronic health evaluation, *ICU* intensive care units, *NGAL* neutrophil gelatinase-associated lipocalin

**Table 4:** Odds ratio by FR in the high NGAL population

	FR positive	FR negative	Odds ratio (95% CI)
AKI stage 3 at 1 week	4/36 (11%)	13/15 (86.7%)	52 (8.5–319.5)
RRT	2/36 (5.6%)	6/15 (40%)	11.3 (2.0–65.9)
AKI stage 3 or death at 1 week	4/36 (11%)	14/15 (93.3%)	112 (11.5–1094.4)

*AKI* acute kidney injury, *FR* furosemide responsiveness

## Discussion

AKI progression frequently occurs in ICU in the context of multiple organ failure [33] and is significantly associated with high mortality in different cohorts of ICU and in patients who have undergone cardiac surgery and those with cardiorenal syndrome [10, 34, 35]. The identification of the potential AKI progression may allow us to initiate early interventions (e.g., more invasive monitoring and RRT) before the development of life-threatening complications. In addition, with the likely development of novel therapies for AKI, an accurate prediction of AKI progression may help to determine patients at the highest risk and those most likely to benefit from such treatment. Several emerging AKI biomarkers, including TIMP-2/IGFBP-7, IL-18, and plasma NGAL, have been demonstrated to predict AKI progression [10, 34]. In particular, TIMP-2/IGFBP-7 was validated for early AKI risk stratification in critically ill patients in multicenter studies [18, 36, 37]. These cell-cycle arrest biomarkers are expected to help in the early detection of patients at risk of AKI in various clinical settings. Recently, FST was suggested to be a significant predictor of progression to AKI stage 3 in patients with AKI stage 1 or 2 [23]. Moreover, Koyner et al. [21] reported a superior efficacy of FST than urinary AKI biomarkers for the prediction of AKI progression. This study demonstrates that both FR and plasma NGAL levels could successfully predict AKI progression as shown by previous studies described above. The novel findings of this study are as follows: (1) FST, as described originally, requires a standardized intravenous furosemide dose of 1 mg/kg. However, this study demonstrated that response to a variable dose of frusemide could also predict AKI progression under actual clinical conditions, when different doses of furosemide were chosen based on patient condition; (2) FR could predict AKI progression in patients with high plasma NGAL levels, while few patients with low plasma NGAL levels exhibited AKI progression. These results indicate that both functional (furosemide response) and structural evaluations (plasma NGAL level) in AKI may be helpful for the prediction of AKI progression.

As described above, FR (mL/mg/2 h) in this study was determined by the 2-h total urine output (mL) following furosemide administration divided by the dose of furosemide (mg). It is well known that the effect of loop diuretics is dose dependent [38]. The delivery of furosemide to the thick ascending limb of the loop of Henle depends on the secretion from the proximal tubular epithelial cells. Because the rate of delivery to the site of action is the most important determining factor for natriuresis induced by furosemide administration, FR in this study might reflect the proximal tubule function even with variable furosemide doses. In the studies involving normal healthy subjects, 10 mg furosemide produced diuresis and 40 mg intravenously administered was associated with the maximal effect. In oliguric AKI, the dose with the maximum effect of furosemide is assumed to be as high as 500 mg [39]. In this study, the furosemide dose ranged from 10 to 340 mg. Our findings provide useful information to clinicians as FR calculated with different doses of furosemide can be used to predict AKI progression. This is because furosemide dosing should be individually determined based on patient condition in a clinical setting.

Another important issue regarding the physiology of furosemide is serum albumin concentration. Hypoalbuminemia results in lower oncotic pressure and fluid shift to the interstitial compartment, which may depress fluid excretion by the kidneys. Previously, colloid infusion with loop diuretics was shown to increase urine output and lower net fluid balance in critically ill patients with hypoalbuminemia and fluid overload [40, 41]. In contrast, despite a possible role of albumin in furosemide-induced diuresis, serum albumin levels were not different between the FR-positive and FR-negative patients in the population with high NGAL levels. Thus, serum albumin levels seemed to have little impact on the response to furosemide.

**Fig. 5:**
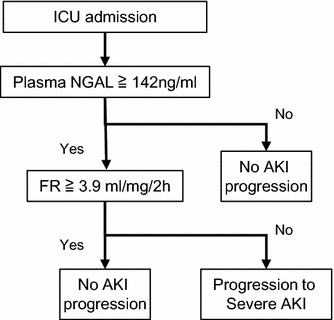
Algorithm of plasma NGAL level and furosemide responsiveness for AKI progression

An ideal AKI biomarker should aid in determining the degree of damage and functional changes in the kidney and help to adequately manage AKI and initiate RRT when needed [22]. Emerging AKI biomarkers, including NGAL, L-FABP, IL-18, and TIMP-2/IGFBP7, are reported to be useful for the early detection of AKI and prediction of progression because AKI impacts the metabolism and excretion of these biomarkers that are produced, excreted, or reabsorbed in the renal tubules [14, 18, 37, 42–47]. However, these biomarkers may be insufficient for the measurement of residual function of the kidney because they monitor damage but not severity of impairment of kidney function [48]. Therefore, we suggest a two-step approach for the prediction of AKI progression: (1) the evaluation of structural damage by plasma NGAL and (2) the subsequent functional assessment by FR (Fig. 5).

This study has several limitations. First, this was a retrospective observational study and the number of patients included in this single-center study was small. Among 523 patients, only 153 were analyzed because furosemide administration was determined based on the clinical situation. In addition, plasma NGAL levels had to be measured on the same day as furosemide administration for study enrollment. Although we showed the significance of FR for predicting severe AKI progression, unmeasured factors could have biased our results. Therefore, inherent bias should be carefully considered while interpreting this study. However, it should be stated that the preliminary findings of this study may suggest that a novel approach combining structural damage makers and functional evaluation may be useful for predicting AKI progression. Future multicenter prospective studies with larger cohorts should be conducted to validate our strategy and findings. Second, the plasma NGAL cutoff level in this study was retrospectively determined and could not be extrapolated in other cohorts. A prospective cohort analysis is required to confirm our results. Third, furosemide was administered based on clinician judgment and criteria for administration depended on clinicians’ decision and the criteria to administer furosemide were vague. Although 1 mg/kg furosemide was used in the original paper for FST [21, 23], furosemide dose administered in our study was different in each patient and was determined by the physician based on the patient’s condition. Again, a prospective study with a predefined furosemide administration protocol is necessary. Finally, we did not evaluate the long-term outcomes in this study. Recent clinical reports demonstrate that AKI has a significant impact on mortality and the progression of kidney disease (e.g., chronic kidney disease or end-stage kidney disease) [49–51]. Further investigation is necessary to determine whether the combination of FR and AKI biomarkers is significant for predicting long-term AKI-related outcomes.

## Conclusions

This retrospective study demonstrated that FR and plasma NGAL may be significant predictors of severe AKI progression in general ICU patients. In addition, FR could predict AKI progression even in patients with high NGAL values, indicating that the sequential evaluation with FR and plasma NGAL could identify patients at a high risk for the development of severe AKI. Of note, identifying high-risk patients may enable to decrease potential adverse effects of furosemide. Finally, careful consideration is necessary before applying the findings of this small retrospective study to clinical practice.

## Additional file

**Additional file 1:** **Figure S1**. Weight-adjusted FR in AKI progression. The boxplots show the differences in weight-adjusted FR between patients (a) without and with the progression to AKI stage 3 and (b) without and with the progression to AKI stage 3 or death within one week. *, p < 0.01. **Figure S2**. prediction of AKI progression by weight-adjusted FR. Receiver operating characteristic curves (ROC) in (a) progression to AKI stage 3 and (b) progression to AKI stage 3 or death at one week.

## Abbreviations

AKI: acute kidney injury
AUC: area under the curve
FR: furosemide responsiveness
FST: furosemide stress test
ICU: intensive care unit
IGFBP7: insulin-like growth factor-binding protein 7
IL-18: interleukin 18
KDIGO: Kidney Disease: Improving Global Outcomes
L-FABP: L-type fatty acid binding protein
NAG: *N*-acetyl-β-d-glucosaminidase
NGAL: neutrophil gelatinase-associated lipocalin
ROC: receiver operating characteristic
RRT: renal replacement therapy
TIMP-2: tissue inhibitor of metalloproteinases
