# Supplementary material for: Response to different furosemide doses predicts AKI progression in ICU patients with elevated plasma NGAL levels
Source: Ann Intensive Care. 2018 Jan 17;8:8. doi: 10.1186/s13613-018-0355-0 (PMC5772346; doi:10.1186/s13613-018-0355-0)
Supplement: Supplementary file 1 — Additional file 1: Figure S1. Weight-adjusted FR in AKI progression. The boxplots show the differences in weight-adjusted FR between patients (a) without and with the progression to AKI stage 3 and (b) without and with the progression to AKI stage 3 or death within one week. *, p < 0.01. Figure S2. prediction of AKI progression by weight-adjusted FR. Receiver operating characteristic curves (ROC) in (a) progression to AKI stage 3 and (b) progression to AKI stage 3 or death at one week. [file 13613_2018_355_MOESM1_ESM.pptx]

## Slide 1
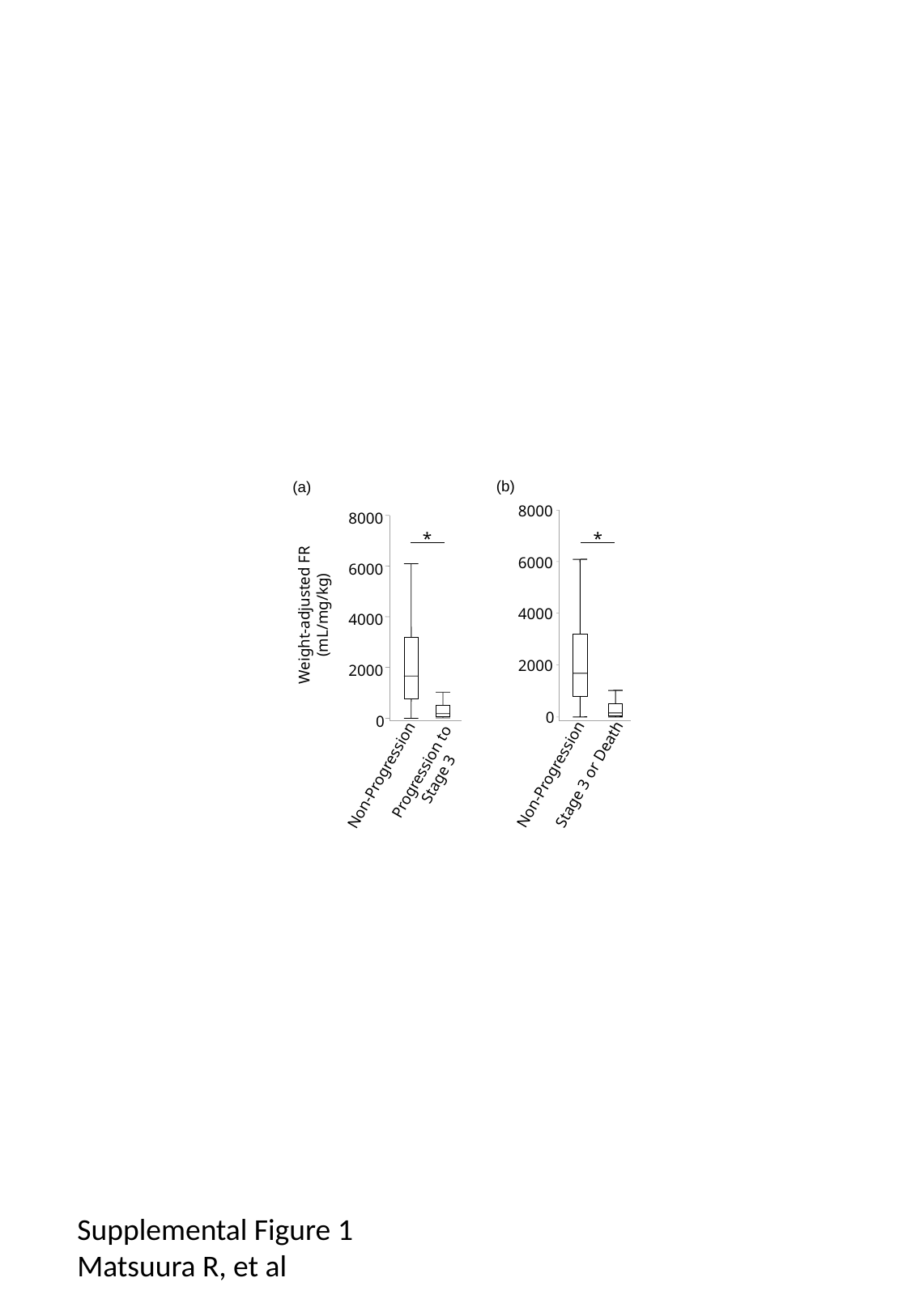

(b)
(a)
8000
8000
*
*
6000
6000
Weight-adjusted FR
(mL/mg/kg)
4000
4000
2000
2000
0
0
Progression to
Stage 3
Non-Progression
Stage 3 or Death
Non-Progression
Supplemental Figure 1
Matsuura R, et al

## Slide 2
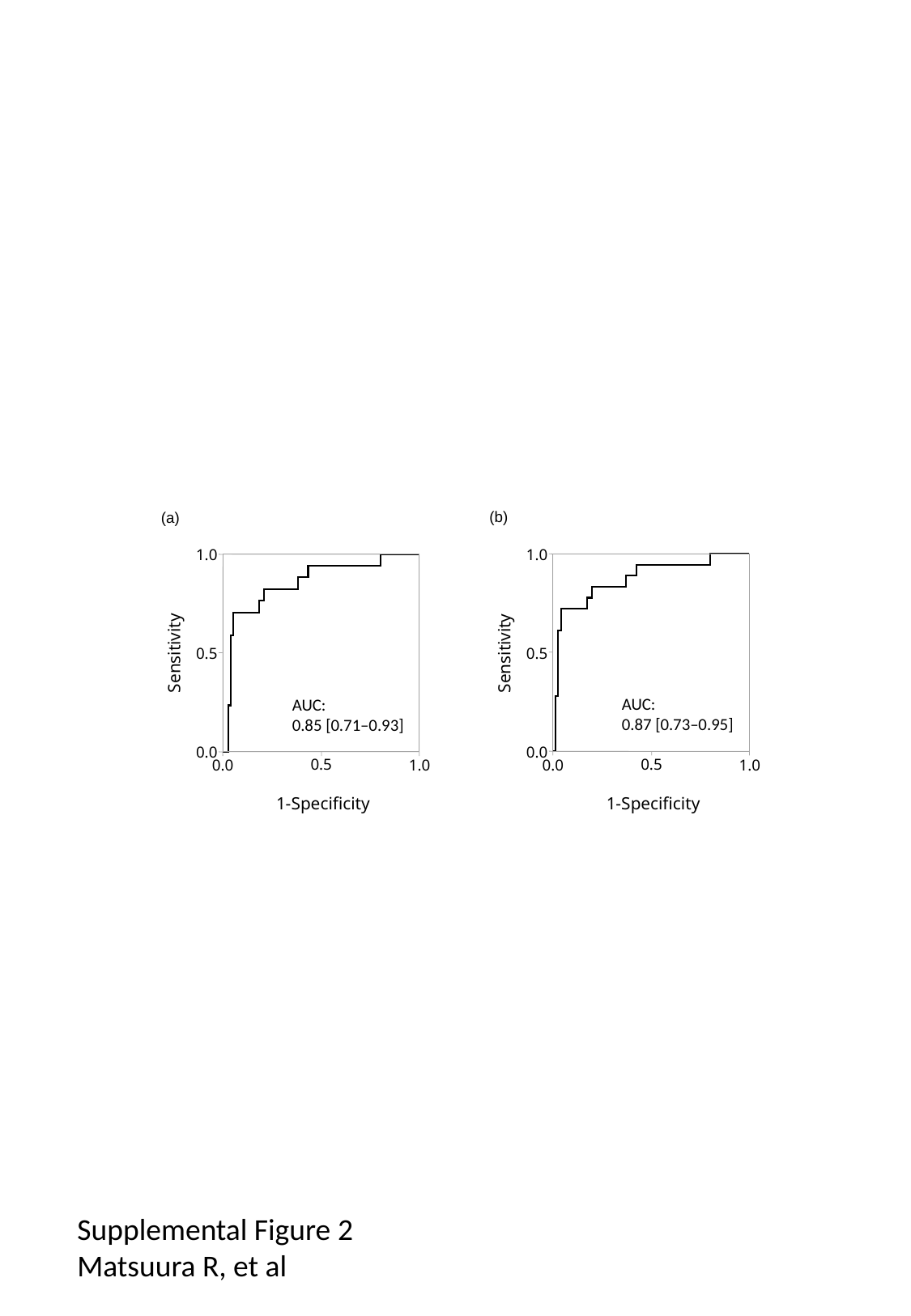

(b)
(a)
1.0
Sensitivity
0.5
0.0
0.5
0.0
1.0
1-Specificity
1.0
Sensitivity
0.5
0.0
0.5
0.0
1.0
1-Specificity
AUC:
0.87 [0.73–0.95]
AUC:
0.85 [0.71–0.93]
Supplemental Figure 2
Matsuura R, et al
